# Photodistributed Stevens–Johnson syndrome and toxic epidermal necrolysis: a systematic review and proposal for a new diagnostic classification

**DOI:** 10.1186/s40001-023-01142-2

**Published:** 2023-06-12

**Authors:** Blake Jeffrey McKinley, Mitchell Edger Allen, Nicole Michels

**Affiliations:** 1 Department of Internal Medicine, Mayo Clinic, 4500 San Pablo Rd S, Jacksonville, FL 32224 USA; 2 Department of Primary Care, Rocky Vista University College of Osteopathic Medicine, Ivins, UT 84738 USA; 3 Department of Medical Humanities and Biomedical Sciences, Rocky Vista University College of Osteopathic Medicine, 8401 S. Chambers Road, Parker, CO 80134 USA

**Keywords:** Stevens–Johnson syndrome, Toxic epidermal necrolysis, Ultraviolet, Photodistributed, Photo induced, Photosensitivity, Sun, Systematic Review

## Abstract

**Background:**

Ultraviolet radiation (UVR) exposure is commonly reported as a risk factor for Stevens–Johnson syndrome (SJS) and toxic epidermal necrolysis (TEN). However, minimal evaluation of photo-induced SJS/TEN has been conducted. Thus, this review identifies all cases of SJS/TEN that are linked to an acute exposure of UVR and outlines the unifying characteristics of these cases. Furthermore, the theoretical pathogenesis, differential diagnoses, and proposed diagnostic criteria are defined.

**Methods:**

PubMed, Google Scholar, and other databases and websites were searched from inception to September 2021 to identify studies that met inclusion criteria. The following keywords were utilized: “Stevens-Johnson syndrome” and “toxic epidermal necrolysis” with “ultraviolet,” “photodistributed,” “photo-induced,” “photosensitivity,” and “photo.” One reviewer assessed study characteristics, with confirmation by a second. The risk of bias was assessed independently by another.

**Results:**

Thirteen patient cases were identified, all reporting ultraviolet radiation prior to rash onset and an underlying causal drug. Case classifications included 7/13 SJS and 6/13 TEN. All cases described the rash as photodistributed with UVR exposure prior to rash onset (delay of 1–3 days) and a causal drug. 10 cases provided evidence that the photodistributed rash lacked linear demarcation (as in a sunburn) with satellite target-like lesions. No cases described a flu-like prodrome.

**Discussion:**

Mucositis, palmar and plantar rash, a positive Nikolsky sign, and a prolonged disease course can help distinguish from photosensitive reactions, while a negative direct immunofluorescence test is important to distinguish from other photo-induced disorders.

**Conclusion:**

Physicians should be aware that UVR may precipitate SJS/TEN in patients taking susceptible drugs. After a 24-h delay from UVR exposure, a non-distinct, photodistributed rash appears with no flu-like prodrome and progresses for at least 48 h to include vesiculobullous eruptions and mucous membrane involvement. Photodistributed SJS/TEN appears to be photo-drug-induced with a unique onset and rash presentation that should be recognized as a distinct diagnosis.

**Supplementary Information:**

The online version contains supplementary material available at 10.1186/s40001-023-01142-2.

## Introduction

Stevens–Johnson syndrome (SJS) and toxic epidermal necrolysis (TEN) are rare cutaneous reactions with epidermal necrosis, vesiculobullous formation, epidermal detachment, and mucous membrane involvement. The classification is determined by the percentage of body surface area (BSA) with epidermal detachment: SJS  < 10%, SJS/TEN overlap 10–30%, and TEN  > 30 [1]; collectively referred to in this study as SJS/TEN. Mortality in SJS/TEN may be as high as 30% [2].

Adverse drug reactions are the most common causes of SJS/TEN with over 100 drugs recognized as causative agents [3]. Ultraviolet radiation (UVR) or ultraviolet light exposure is commonly reported as a physical risk factor for SJS/TEN in book chapters [4–6], review studies [7–13], case reports [14–20], and the point-of-care medical resource, UpToDate [21]. However, the connection between UVR and SJS/TEN is poorly documented. Since no systematic review has been conducted, the current evidence exists as individual case reports. One of the first cases to report the link between UVR and TEN was published in 1996 and has been cited, according to Google Scholar, 71 times [22].

The purpose of this review is to identify all cases of SJS/TEN that are linked to UVR and outline the unifying characteristics of these cases while comparing and contrasting the findings with traditional SJS/TEN cases [1–3]. This will provide an in-depth analysis of the implications of UVR concerning SJS/TEN cases. Additionally, a comprehensive review of the theoretical pathogenesis, important differential diagnoses, and proposed diagnostic criteria will allow guidance for clinicians to understand, diagnose, and correctly treat future cases of photodistributed SJS/TEN, the diagnosis that the authors propose calling this disorder.

It is important for physicians to recognize the distinguishing factors of photodistributed SJS/TEN as many conditions present similarly, such as photosensitivity reactions, that generally do not require intensive treatment. Recognizing a presentation of photodistributed SJS/TEN can help the patient promptly obtain the critical care they need.

## Methods

### Data sources and searches

Databases, registers/repositories, and websites/web search engines were searched for relevant studies from inception to the reported search dates. The following databases were searched: PubMed (searched 09/09/2021), TRIP medical database (searched 09/10/2021), OECD (searched 09/13/2021), OAIster: Find the Pearls (searched 09/13/2021), New York Academy of Medicine Grey Literature Report (searched 09/13/2021), Cochrane Library (searched 09/14/2021), Qinsight (searched 09/15/2021). The following registers/repositories were searched: ClinicalTrials.gov (searched 09/13/2021), Sigma Repository (searched 09/13/2021), Social Science Research Network (SSRN) (searched 09/13/2021), National Technical Information Service (NTIS) (searched 09/13/2021). The following websites/web search engines were searched: Google Scholar (https://scholar.google.com/ searched 09/10/2021), Grey Matters: a practical search tool for health-related grey literature (https://www.cadth.ca/grey-matters-practical-tool-searching-health-related-grey-literature-0/ searched 09/13/2021), Bielefeld Academic Search Engine (BASE) (https://www.base-search.net/ searched 09/15/2021), USA.gov (https://www.usa.gov/ searched 09/15/2021), Medicine Case Reports and Protocols (https://journals.lww.com/md-cases/pages/default.aspx searched 12/29/2021), Medscape (https://www.medscape.com/ searched 09/16/2021), UpToDate (https://www.uptodate.com/ searched 9/16/2021). The first 150 results of each search were screened for inclusion in Google Scholar, USA.org, and Qinsight if individual searches produced more than 150 results. Studies were searched in English; however, studies that were published in languages other than English were translated and evaluated in the same manner as English-language studies. For each search, the following keywords were used: “Stevens–Johnson syndrome” and “toxic epidermal necrolysis” with “ultraviolet,” “photodistributed,” “photo-induced,” “photosensitivity,” and “photo.” UpToDate was searched using the following keywords: “Stevens–Johnson syndrome” and “toxic epidermal necrolysis.” The full strategies are shown in Additional File 1.

### Study selection

Inclusion criteria required each study to contain a formal diagnosis of SJS or TEN with a reported etiology (cause). An identified cause, most commonly in SJS/TEN being a drug, was necessary to support a SJS/TEN diagnosis. The cause of the SJS/TEN disorder also needed to be active prior to UVR exposure, but UVR exposure needed to occur prior to SJS/TEN onset. For example, a patient who is exposed to UVR between the ingesting of a causal drug and the onset of SJS/TEN would qualify for the study. This timeline is important to support that the acute exposure of UVR contributed to the patient’s SJS/TEN. Furthermore, an active potential cause of SJS/TEN was necessary prior to rash onset as UVR exposure itself has not been reported to cause SJS/TEN. We excluded any case that merely mentioned the possibility of SJS or TEN as a diagnosis. Cases that included a diagnosis of erythema multiforme were also excluded.

Many studies of patients with underlying lupus erythematosus (LE) reported SJS/TEN-like reactions linked to drug use and rashes presenting after UVR; however, most of these cases recognize lupus erythematosus as the causative factor for the rash presentation rather than clearly diagnosing SJS/TEN [23–27]. Furthermore, many of these cases are recognized under a relatively new diagnosis: TEN-like acute cutaneous LE [23, 27]. Although a definitive rule-out of SJS/TEN-like LE would be preferred for all cases included in this review, many cases did not provide a complete diagnostic workup in the case presentation, many times providing only positive results. (See Table 1 to review the case specific details that were provided for SJS/TEN-like LE rule-out of the cases included in this review [22, 28–39]). Thus, cases of SJS/TEN were excluded when the patient reported a new onset or history of LE, but evidence of a definitive rule-out of SJS/TEN-like LE for each case was not part of the criteria for inclusion.

**Table 1:** Case specific details for SJS/TEN-like LE rule-out

Reference numbers	Suggestive SJS/TEN-like LE rule-out	Definitive SJS/TEN-like LE rule-out
[28]	N/A	N/A
[22]	N/A	Negative direct immunofluorescence for immunoglobulins and complement
[29]	Normal complement concentrations (C3, C4)	Negative antinuclear antibody
[30]	Normal complement concentrations (C3, C4) Previously diagnoses of seronegative symmetrical polyarthritis	N/A
[31]	N/A	N/A
[32]	Diagnosed with Sjogren’s syndrome 7 years prior, confirmed with serology and a labial biopsy No history of sensitivity to sunlight	Negative direct immunofluorescence for immunoglobulins and complement Negative antibodies to double-stranded DNA. (Positive antinuclear antigen at a dilution of 1: 4000 with speckled pattern and positive anti-Ro and anti-La antibodies.)
[33]	Multi-specialty workup: included dermatology, infectious disease, and plastic surgery	N/A
[34]	Multi-specialty workup: Evaluated and treated in burn unit and dermatology, ophthalmology and gynecology services were consulted	N/A
[35]	N/A	N/A
[36]	N/A	Negative direct immunofluorescence Negative antinuclear antibodies
[37]	N/A	N/A
[38]	N/A	Negative direct immunofluorescence
[39]	N/A	Negative antinuclear antibody Negative anti-Smith antibody Negative anti-DNA antibody

Authors interpretation of evidence for ruling out SJS/TEN-like LE is as follows: a negative direct immunofluorescence is observed in photodistributed SJS/TEN, whereas it is positive in SJS/TEN-like LE. A negative antinuclear antibody has a strong negative predictive value for ruling-out LE. If ANA is positive but anti-Smith Antibody or double-stranded DNA Antibody is negative, LE is unlikely. In this study, cases that report a negative direct immunofluorescence or a negative lupus specific autoimmune workup are considered definitive rule out of SJS/TEN-like LE. Additional suggestive findings: normal serum C3 and C4 suggests absence of SLE, active SLE often results in decreased compliment proteins; multispecialty work-up, suggesting that further workup was performed but not reported; and previous rheumatological diagnosis, suggesting that SLE would have been ruled out at the time of previous diagnosis

*SJS*   Steven’s-Johnson Syndrome, *TEN*   toxic epidermal necrolysis, *LE*   lupus erythematosus, *N/A* not available

**Fig. 1:**
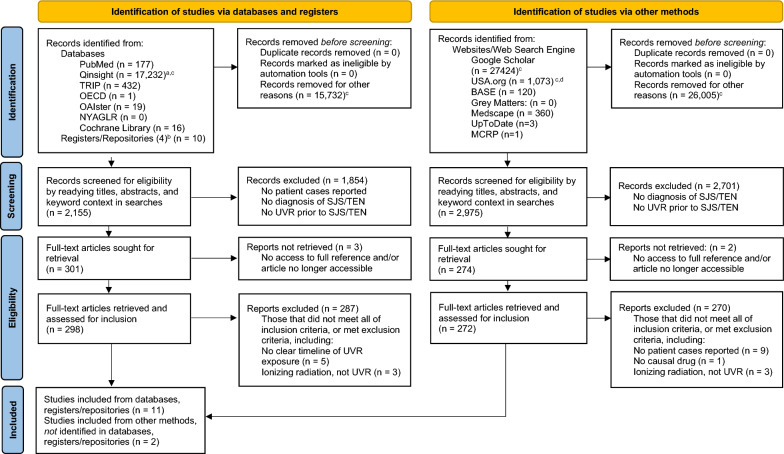
Study Selection Process to Identify Cases of Photodistributed Steven’s–Johnson Syndrome and Toxic Epidermal Necrolysis. **a** Focused searches were first looked at before broader results. As focused results found fewer and no unique articles to be included in the study, only broader results were computed in the analysis. **b** Additional registers/repositories searched: National Technical Information Service (NTIS), Social Science Research Network (SSRN), ClinicalTrials.org, Sigma Repository. **c** Searches that produced > 150 results, the first 150 were screened for inclusion and others removed. **d** USA.org did not report total results for each search, but first 150 results of each search were screened (those with > 150). *TRIP*  TRIP medical database. *OAIster* OAIster: Find the Pearls, *NYAGLR*   New York Academy of Medicine Grey Literature Report, *SJS*   Steven’s–Johnson syndrome, *TEN*  Toxic epidermal necrolysis, *BASE*   Bielefeld Academic Search Engine, *Grey Matters *  Grey Matters: a practical search tool for health-related grey literature, *MCRP*  Medicine Case Reports and Protocols, *UVR *  Ultraviolet radiation. Table modified from: Page MJ, McKenzie JE, Bossuyt PM, Boutron I, Hoffmann TC, Mulrow CD, et al. The PRISMA 2020 statement: an updated guideline for reporting systematic reviews. BMJ 2021;372:n71. https://doi.org/10.1136/bmj.n71

This was a pre-planned search with the objective to locate all available studies that align with the inclusion criteria, including abstracts. No studies were discarded based on publication date. One reviewer determined if the studies met inclusion criteria by reading titles and abstracts and performing a full-text evaluation before including a study in the review (Fig. 1). Another reviewer then confirmed study inclusion. The risk of bias was assessed by a third investigator via an independent analysis to determine if the studies met the inclusion criteria.

### Data extraction and quality assessment

Case-specific details were identified for each case report. The case details included the associated patient demographics of age and sex (race was not reported as only two cases provided this information); type of case: SJS, SJS/TEN, or TEN; causal drug for SJS/TEN cases with the corresponding diagnosis for which it was prescribed; UVR exposure amount and location; time from UVR exposure to rash onset; reported timeline of progression/worsening of rash; whether the rash was described as photodistributed; whether the rash extended to clothing covered skin; whether a flu-like prodrome was reported; initial signs/symptoms; SJS/TEN diagnostic specific details such as mucus membrane involvement, biopsy results, palmar and plantar rash, Nikolsky sign results; and treatment (Table 2). One reviewer evaluated each study to identify case details by performing a full-text evaluation. Minimal interpretation was required to extract data or case details for each category except for “reported flu-like prodrome” and “rash extension to clothing covered skin.” Flu-like prodrome was evaluated as follows: the finding was considered negative if the initial symptoms that were reported did not include characteristic findings of a flu-like prodrome (malaise, fever) *prior* to rash onset. Rash extension to clothing covered skin was evaluated by reviewing the images that were provided in each case report. Images were evaluated for the lack of linear demarcation between UVR exposed skin and clothing covered skin (not a sharp line as is observed in a common sunburn) and satellite lesions (target-like lesions that are isolated from the rest of the rash on clothing covered skin). Findings consistent with either of these criteria were considered positive. Once data were extracted, it was confirmed by another. The risk of bias was assessed independently by a third investigator.

**Table 2:** Individual case results of photodistributed Stevens–Johnson Syndrome and toxic epidermal necrolysis

Reference to case	[28]	[22]	[29]	[30]	[31]	[32]	[33]	[34]	[35]	[36]	[37]	[38]	[39]
Study year	1989	1996	2000	2003	2005	2008	2010	2011	2014	2016	2018	2021	2021
Study type: case report (C), abstract (A)	C	C	C	C	C	C	C	C	C	C	A (poster abstract)	C	C
Demographics (age, gender)	12, M	23, F	16, F	34, M	66, M	29, F	30, M	22, F	19, F	48, F	65, F	22, F	18, F
SJS or SJS/TEN or TEN	SJS	TEN	SJS	SJS	TEN	TEN	TEN	TEN	SJS	SJS	SJS	TEN	SJS
Causal drug	3 weeks chloroquine and sulfadoxine-pyrimethamine	14 days clobazam	3 weeks of carbamazepine, increased doses from 200 mg/day to 600 mg/day	2 months sulfasalazine	One dose Naproxen Sodium (Rash not present until 3 days after drug exposure. Experienced similar eruption three times after intake of naproxen, in the last 5 years	3 years of hydroxychloroquine (Also used gabapentin for 2 years for postherpetic neuralgia)	19 days of lamotrigine and increased dose of chlorpromazine after 10 years of use (Also used alprazolam, zolpidem, lithium)	One dose (200 mg) of Ibuprofen, not first-time use (1 day after drug use, rash started, stable 2 days, then sever rash after tanning bed)	10 days ciprofloxacin/ 1-day fluconazole (Rash presented day after drug course)	3 days itraconazole (Rash presented day after drug course)	Lamotrigine (duration of use not specified)	10 days of lamotrigine	One dose of tramadol (Only drug taken 7–10 prior to rash onset)
Diagnosis associated with drug	Prophylactic malaria	Alopecia areata on the scalp	Epilepsy (Additional history of allergic rhino-conjunctivitis)	Seronegative symmetrical polyarthritis	Keratitis of the right eye	Sjogren’s syndrome	Bipolar 1	Menstrual discomfort	Vaginal infection	Vaginal yeast infection	Epilepsy	Bipolar disorder	Hip pain
UVR exposure place and amount	Rash presented after first day of sun exposure at the seashore in Thailand	1 day at beach	45 min of sunlight, explained as an intense exposure	N/A	N/A	Sunburnt on Mediterranean cruise	19 days in psychiatric ward, often lay in sun during day	Eight-minute exposure in tanning bed	1 day at beach	Several hours of sun exposure all three days of itraconazole use	N/A	Daily tanning bed use all 10 days of lamotrigine use	1 day at a lake
Reported photodistributed rash (Y/N)	Y	Y	Y	Y	Y	Y	Y	Y	Y	Y	Y	Y	Y
Rash extension to clothing covered skin (Y/N) (lack of linear demarcation, satellite lesions)	Y Lack of linear demarcation, satellite lesions	Y Lack of linear demarcation, satellite lesions	N/A	N/A	Y Lack of linear demarcation, satellite lesions	Y Lack of linear demarcation, satellite lesions	Y Lack of linear demarcation, satellite lesions	Y Lack of linear demarcation, satellite lesions	Y Lack of linear demarcation, satellite lesions	Y Satellite lesions under areola	N/A	Y Lack of linear demarcation, satellite lesions	Y Lack of linear demarcation, satellite lesions
Time from UVR exposure to rash onset	N/A	36 h	1 day	3 days	N/A	N/A	N/A	1 day (to change from baseline rash)	2 days	1 day	N/A	N/a	1 day
Timeline of progression/ worsening of rash	Reported worsening rash at 3 days	4 days until progression of rash stopped	Progressed past 48 h	On day 12 of hospitalization, skin lesions were improving	Eruption of new lesions ceased on 12^th^ day of admission	14 days (7 days to hospital admission, then 7 days later, skin detachment extended to involve at least 60% of the body surface area)	5 days of gradual progression to painful bullous eruptions and 30% total body surface area	N/A	N/A	Reported worsening rash at 48 h, Presented to ED 5 days	N/A	N/A – Hospitalized for 12 days	Day 5 of rash presented to hospital from continued worsening symptoms
Mucus membrane involvement	Conjunctivitis, erosions of the buccal mucosa and lips, and erosive balanitis	Bilateral conjunctival, oral, and genital mucosa	Oral, ocular, and genital mucosa	Oral and genital mucosa	Buccal and palatal mucosa	Hemorrhagic crusting of the lips, buccal mucosa and tongue. Eyelids were eroded with intense conjunctival Injection. Vulvar and vaginal lesions	Oral mucosa, conjunctivitis	Oral, ocular, and vaginal mucosa	White vesicles and erosions on the lips. Ulcers of the oral mucosa. Erythematous patches within the labia	Irritation and redness of the conjunctivas. Necrotic crusts on the lips. Oral mucosal inflammation and ulcerations	N/A	Conjunctival injection. Bullae involving the vermillion and mucosal lips, buccal mucosa, and tongue. Swelling of genitalia	Cheilitis, bleeding oral ulcers, and bilateral conjunctival hyperemia with purulent discharge
Palmar and plantar rash (Y/N)	Y Iris lesions on palms and soles	N/A	N/A	Y Annular iris lesions on palms of the hands and the soles of the feet	N/A	N/A	N/A	N/A	N	Y After 48 h the palms and soles were erythematous and painful with some blisters between the toes	N/A	Y Pruritic eruption including palms and soles	Y Maculopapular rash of palms of hands and soles of feet
Nikolsky sign ( ±)	N/A	+	N/A	+	+	+	N/A	N/A	N/A	-	N/A	N/A	+
Skin biopsy	N/A	Hydropic and vacuolar degeneration of the basal cells, with exocytosis of mono-nuclear cells in the epidermis and a subepidermal bulla with festooning of the underlying papillary dermis Direct immunofluorescence for immunoglobulins and complement was negative	Presence of a subepidermal blister with necrosis of the epidermal keratinocytes and intense chronic inflammatory infiltrate with some eosinophils around the vessels and hair follicles in the dermis^a^	Full-thickness epidermal necrosis. The cytoplasmic limits of the cells had been lost	Extensive epidermal necrosis; areas around the necrosis showed vacuolar degeneration of the basal layer, edema of the superficial dermis, melanophages and a mild perivascular lymphocytic infiltration	Subepidermal, cell-poor bulla formation with full thickness epidermal necrosis, consistent with TEN Direct immunofluorescence for immunoglobulins and complement was negative	N/A	Lymphocytic infiltrate at the dermal–epidermal junction with some apoptotic keratinocytes consistent with TEN	Intact stratum corneum with interface dermatitis and full thickness epidermal necrosis compatible with SJS	Necrotic epidermis and interface dermatitis with vacuolization Direct immunofluorescence was negative	N/A	Interface dermatitis with scattered dyskeratosis, consistent with SJS/TEN Direct immunofluorescence was negative	Findings consistent with SJS
Reported flu-like prodrome (Y/N) Reported initial signs/symptoms	N Severe erythema confined to sun-exposed areas. Within the next 3 days fever and malaise accompanied the concomitant development of round, annular, concentric typical iris lesions in sun-protected areas	N Pruriginous and erythematous eruptions	N General malaise, fever, conjunctival injection, erythematous and infiltrated lesions (Quadro de malestar general, fiebre, inyeccion conjunctival, lesiones eritematosas e infiltadas)	N Severe cutaneous eruption that had started 3 days after sun exposure, No abdominal pain, nausea, vomiting, or fever was present	N An erythematous and bullous rash had started on his face and neck, which rapidly spread to his arms and legs	N Facial swelling and a painful erythematous eruption with lethargy and anorexia	N Whole body itched and he developed restlessness, then wheal-like erythematous, itching skin rashes	N Four hours following the tanning bed exposure the patient noted increased itchiness of her tanning bed exposed skin. The next morning the patient experienced severe redness, pain, and the beginning of blister formation on her tanning bed exposed abdomen, back, face, and proximal arms	N A red rash concentrated on her chest developed	N Without any prodromes, developed rash on upper trunk	No initial symptoms reported	N Diffusely pruritic eruption involving her upper back and chest that later extended to the face and extremities, including the palms and soles	N Onset of an erythematous burning rash on her shoulders
Treatment	Systemic corticosteroids and antibiotics (4 weeks to recovery)	Pentoxifylline and prophylactic systemic antimicrobials, supportive measures	methyl-prednisolone	N/A	Methylprednisolone	Benzyl penicillin, Flucloxacillin, prophylactic low molecular weight heparin, eye steroid and lubricant	Antibiotics (4 weeks to recovery)	IM Corticosteroid and vancomycin, 2 days I.V. cyclosporine, 3 days of intravenous immunoglobulin G	N/A	3-week course of oral steroids and betamethasone dipropionate ointment	N/A	5-day regimen of oral cyclosporine 5 mg/kg/d	Prophylactic oral doxycycline and topical oral and ocular medications

^a^Original version prior to translation to English: “la presencia de una ampollo subepidérmica con necrosis de los queratinocitos del techo de la misma e intenso infiltrado inflamatorio crónico con algunos eosinófilos alredador de los vasos y folículos pilosos en la dermis”

*SJS*  Stevens–Johnson syndrome, *TEN*  Toxic epidermal necrolysis, *UVR*   Ultraviolet radiation

### Data synthesis and analysis

The approach for data synthesis and analysis was qualitative. The combined data extracted from the studies comprised the results. These results were then compared to traditional SJS/TEN cases to assess for congruent or conflicting findings. Drug causality assessment was determined for each drug-induced case using the algorithm of drug causality for epidermal necrolysis (ALDEN) [40]. No software, coding, or study comparisons were needed for analysis. All studies were equally weighted, as each met the same inclusion criteria. All authors were involved in the synthesis of data and final analysis.

## Results

After a thorough review, 13 total cases were identified, dating from the year 1989 to 2021. The studies consisted of 12 case reports [22, 28–36, 38, 39] and one poster abstract [37]. Study bias is minimal as each study reported objective findings for their corresponding case, providing raw data for our analysis. All cases were drug induced and identified a causal drug with UVR exposure between drug ingestion and disease onset. Patients ranged between 12- and 66-years-old with a female predominance of 9/13 (69.2%). Case classification breakdown was as follows: 7/13 (53.8%) cases were SJS, 0/13 (0%) cases were SJS/TEN overlap, and 6/13 (46.2%) cases were TEN.

The source of the UVR was specified in 10/13 (76.9%) cases: eight reported direct sunlight exposure and two reported artificial UVR exposure from tanning beds. Cases that reported the source of direct sunlight varied in their descriptions: sunburned on cruise, sun-bathed for hours over three consecutive days, rolled around and laid in the grass for 19 consecutive days while hospitalized in the psychiatric ward, exposed to 45 min of intense sunlight, and four spent a day at the beach/lake/seashore. The UVR obtained from a tanning bed was explained as a single eight-minute session in one case, while the other reported daily sessions for 10 days.

Drug regimens that were responsible for the individual SJS cases: three weeks on carbamazepine, two months on sulfasalazine, ten days on ciprofloxacin with one-day on fluconazole, three weeks on chloroquine and sulfadoxine–pyrimethamine, three days on itraconazole, unspecified duration on lamotrigine, and a single dose of tramadol. Drug regimens that were responsible for the individual TEN cases: 19 days on lamotrigine and an increased dose of chlorpromazine after ten years of use, ten days on lamotrigine, 14 days on clobazam, three years on hydroxychloroquine, a single dose of naproxen sodium, and a single dose of ibuprofen. The only repeat causal drug was lamotrigine with three cases: one of SJS and two of TEN. These drugs fall into classes that are known to cause SJS/TEN, including antibiotics, antiepileptics, antimalarials, sulfonamides, and nonsteroidal anti-inflammatory drugs. The ALDEN algorithm revealed 8/13 (61.5%) case specific drugs as very probable for causing SJS/TEN (highest category), 1/13 (7.7%) as probable, 3/13 (23.1%) as possible, and 1/13 (7%) as undetermined due to lack of information (Table 3). No pattern regarding the underlying medical conditions for which the drugs were prescribed was identified.

**Table 3:** ALDEN Drug causality assessment of drugs in individual cases of photodistributed Steven's–Johnson syndrome and toxic epidermal necrolysis (table format modified from Sassolas et al., 2010 [40]

Reference to study	[28]	[22]	[29]	[30]	[31]	[32]	[33]	[34]	[35]	[36]	[37]	[38]	[39]
Delay from initial drug component intake to onset of reaction (index day)^a^	+3	+3	+3	−1	+3^b^	−1	+3	+3^b^	+3	+1	N/A	+3	+3
Drug present in the body on index day^c^	0	0	0	0	0	0	0	0	0	0	0	0	-3
Prechallenge/rechallenge^d^	0	0	0	0	+4	0	0	+1	0	0	N/A	0	0
Dechallenge^e^	0	0	0	0	0	0	0	0	0	0	0	0	0
Type of drug (notoriety)^f^	+3	+3	+3	+3	+3	+3	+3	+3	+3	+3	+3	+3	+2
Other cause^g^	0	0	0	0	0	0	0	0	0	0	0	0	0
Final Score^h^	6	6	6	2	10	2	6	7	6	4	N/A	6	2

^a^Suggestive (5 to 28 days) = +3, Compatible (29 to 56 days) = +2, Likely (1 to 4 days) = +1, Unlikely (>56 days) = −1, Excluded (drug started on or after the index day = −3

^b^In case of previous reaction to the same drug, only changes for: Suggestive (1 to 4 days) = +3 Likely (5 to 56 days) = +1

^c^Definite (Drug continued up to index day or stopped at a time point less than five times the elimination half-life before the index day) = 0; doubtful (Drug stopped at a time point prior to the index day by more than five times the elimination half-life but liver or kidney function alterations or suspected drug interaction are present) = −1; Excluded (Drug stopped at a time point prior to the index day by more than five times the elimination half-life, without liver or kidney function alterations or suspected drug interactions) = −3

^d^Positive specific for disease and drug (SJS/TEN after use of same drug) = 4; Positive specific for disease or drug (SJS/TEN after use of similar drug or other reaction with same drug) = 2; Positive unspecific (Other reaction after use of similar drug) = 1; Not done/unknown (No known previous exposure to this drug) = 0; Negative (Exposure to this drug without any reaction, before or after reaction) = −2

^e^Neutral (Drug stopped or unknown) = 0; Negative (Drug continued without harm) = −2

^f^Strongly associated (Drug of the “high-risk” list according to previous case–control studies) = 3; Associated (Drug with definite but lower risk according to previous case–control studies) = 2; Suspected (Several previous reports, ambiguous epidemiology results - drug “under surveillance”) = 1; Unknown (All other drugs including newly released ones) = 0; Not suspected = −1 (No evidence of association from previous epidemiology study with sufficient number of exposed controls)

^g^Possible (Rank all drugs from highest to lowest intermediate score. If at least one has an intermediate score >3, subtract 1 point from the score of each of the other drugs taken by the patient. “another cause is more likely”) = −1

^h^Final Score: Range: -12 - 10; Very unlikely <0, unlikely 0–1, possible 2–3, probable 4–5, very probable ≥6

For further details on how to assign scores, see “Table 5 Details of the algorithm of drug causality for epidermal necrolysis (ALDEN)” from Sassolas et al. [40]

*SJS*  Stevens–Johnson syndrome, *TEN*  toxic epidermal necrolysis,* ALDEN*  algorithm of drug causality for epidermal necrolysis

Further analysis revealed that 13/13 (100%) cases described the rash presentation to be in a photodistributed pattern, largely affecting the cutaneous areas exposed to direct sun with minimal rash involvement of the regions covered by a swimsuit or clothing. Ten cases provided images to evaluate if the rash extended to clothing covered skin, all positive. In six cases, the time to rash onset after UVR exposure was specified, ranging from 1 to 3 days after sun exposure. A rash was described as either the initial sign ± concomitant signs/symptoms in 10/13 cases, while 2/13 cases described experiencing itchiness that was followed quickly by rash onset and one case did not provide a description of initial signs/symptoms. Thus, no cases described a flu-like prodrome prior to rash onset. Timeline of the progression/worsening of rash was reported as continuing for at least 48 h and reported evidence suggested that it lasted for 12–14 days in multiple cases. Mucous membrane involvement was mentioned in twelve cases: 12/12 (100%) with oral mucosa ± lip involvement, 9/12 (75%) with conjunctival involvement, and 8/12 (66.7%) with genital involvement. Nikolsky’s sign was positive in the 5/6 (83.3%) cases in which it was mentioned. Six cases described findings of palmar and plantar surfaces: 5/6 (83.3%) rashes were present. Skin biopsy was included in 10/13 (76.9%) case reports, all consistent with SJS/TEN. Findings of direct immunofluorescence (DIF) were reported in 4/10 (44.4%) biopsies; all four cases were negative. Treatment was specified in 10/13 (76.9) cases. Of those, 5/10 (50%) involved systemic steroids, 6/10 (60%) involved antibiotics, 2/10 (20%) involved cyclosporine, and 1/10 (10%) involved intravenous immunoglobulin G. As a part of treatment, all offending drugs were discontinued and there were no indications that any were restarted, including drugs used to treat chronic medical conditions such as carbamazepine, sulfasalazine, hydroxychloroquine, and lamotrigine. All patients recovered (Table 4).

**Table 4:** Summarized results of Photodistributed Stevens–Johnson syndrome and toxic epidermal necrolysis

13 total cases: 12 case reports [22, 28–36, 38, 39] and 1 poster abstract [40]
Case classifications: SJS 7/13 (53.8%), SJS/TEN overlap 0/13 (0%), and 6/13 (46.2%)
Patients ranged between 12- and 66-year-old with a female predominance of 9/13 (69.2%)
All cases described rash presentation as photodistributed
All cases reported ultraviolet radiation prior to rash onset
All cases recognized a causal drug
All patients recovered
10 cases reported source of the UVR: 8 cases from direct sunlight, 2 cases from tanning bed
10 cases provided images of rash extension to clothing covered skin (lack of linear demarcation, satellite lesions)
10 cases described rash progression/worsening > 48 h (up to weeks)
12 cases report various mucous membrane involvement, all with oral mucositis
9 cases included biopsy findings, all consistent with SJS/TEN (4 cases reported direct immunofluorescence findings, all negative)
7 cases reported time from UVR exposure to rash onset, all 1–3 days later
6 cases reported Nikolsky sign findings, 5 positive
6 cases reported palmar and plantar findings, 5 positive for rash
No cases reported an influenza-like prodrome

*SJS* Stevens-Johnson syndrome, *TEN *  toxic epidermal necrolysis, *UVR * Ultraviolet Radiation

## Discussion

### The role of UVR in photodistributed SJS/TEN

The nature of the rash in these cases provides evidence that UVR precipitates SJS/TEN in patients taking susceptible drugs. Rash timing occurred after sun exposure and originated on sun-exposed areas of the body. As the rash progressed over time to form vesiculobullous lesions with skin sloughing, the photodistributed pattern remained. It is the photodistributed pattern that led the authors to name these disorders photodistributed SJS, photodistributed SJS/TEN overlap, and photodistributed TEN, referred to collectively in this study as photodistributed SJS/TEN. Furthermore, a unique finding in this study is that no cases describe a flu-like prodrome, which generally proceeds rash onset by approximately 3 days in SJS/TEN [1, 41]. Photo-induced cases generally describe the rash as their primary manifesting symptom of their disorder. As traditional cases of SJS/TEN do not manifest initially with a rash, but a flu-like prodrome, and do not present in a photodistributed pattern, this is evidence that UVR precipitates the disease process, altering its course and clinical presentation.

**Fig. 2:**
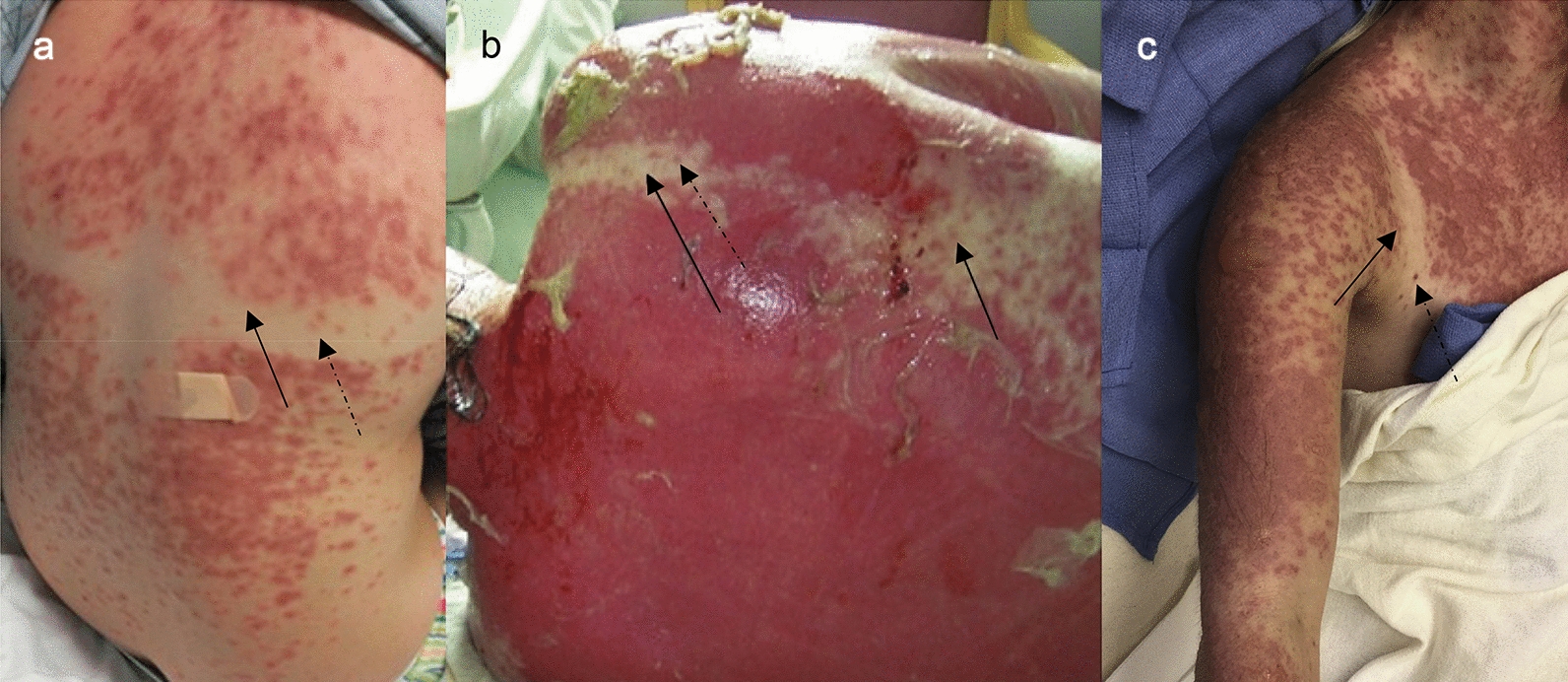
Rash in photodistributed Steven’s–Johnson Syndrome and Toxic Epidermal Necrolysis [32, 35, 38]. All three images were obtained from cases in this review and demonstrate a photodistributed pattern with the sparing of skin covered by bikini swimsuits. The rash distribution is less demarcated (solid arrows) without sharp lines as is observed in a common sunburn, and with satellite lesions (dotted arrows) that are target-like and isolated from the rest of the rash in areas where the swimsuit covered the skin during the UVR exposure. The images were from patient cases of **a** Steven’s–Johnson syndrome from 10 days of ciprofloxacin and sun exposure at the beach [35], **b** toxic epidermal necrolysis from 3 years of hydroxychloroquine and sun exposure on a cruise [32], and **c** toxic epidermal necrolysis from 10 days of lamotrigine and tanning bed exposure [38]

Further evaluation of the rash reveals that the photodistributed rash presented after a minimum of 24 h from UVR exposure with non-discrete borders (less demarcated than a sunburn) and satellite lesions on UVR protected (clothing covered) skin (Fig. 2). Furthermore, the rash commonly spreads to the palmar surfaces of the hands and the plantar surfaces of the feet, areas that are reported as receiving less UVR exposure compared to other areas of the body [42, 43]. The delayed onset of the rash, with extension onto areas with less UVR exposure, along with the observed finding of inflammation and ulcerations of mucous membranes, supports that UVR triggered a systemic immune response that is consistent with SJS/TEN, even though sun-exposed areas remained the most severely affected.

Additional evidence that elucidates the role of UVR in SJS/TEN cases was presented in a Chilean study after an analysis was performed on 24,521,796 hospital discharges nationwide [44]. The study revealed that the incidence of SJS/TEN occurred with an increased frequency in regions of the country with higher altitude, leading to the conclusion in the study that the additional UVR exposure at high altitudes may play a role. The photodistributed rash, the altered primary manifesting symptoms (rash vs. flu-like prodrome), the evidence of a systemic immune response that remains most prominent in sun exposed areas, and the increased incidence of SJS/TEN in areas of increased UVR exposure all suggest that UVR precipitates the onset and clinical presentation of photodistributed SJS/TEN cases.

### Causal drug

All cases that were included in this review reported a causal drug, even though the search criteria did not require a causal drug. Additionally, no cases reported that UVR caused SJS/TEN by itself. Therefore, it is likely that the drug plays a key role in the pathogenesis of photodistributed SJS/TEN. It is postulated that the direct effect of UVR on the drug itself, or the surrounding cutaneous tissue that then affects the drug, leads to the sequelae of the SJS/TEN presentation (see 4.3 Pathogenesis Theories of Photodistributed SJS/TEN). Thus, it is important to validate that the reported drugs were responsible for their cases of photodistributed SJS/TEN. Drug causality assessment tools (CAT) are limited in their ability to determine culprit drugs in SJS/TEN cases. Their findings have been shown to vary between investigators [45]. Consequently, the results should always be compared to a thorough clinical assessment. If a drug CAT is to be employed in a SJS/TEN case, the ALDEN CAT is arguably the most reliable [45]. In this analysis, all cases, apart from one with insufficient information, revealed that the identified drugs were very probable, probable, or possible for causing SJS/TEN. These results, along with a complete review of each case, revealed that the identified drugs could have reasonably acted as the culprit drugs.

### Pathogenesis theories of photodistributed SJS/TEN

Due to technological advancement and continued research, our understanding of the pathogenesis of SJS-TEN continues to be better understood. Drugs are implicated as the causative agent in as many as 80% of cases with anticonvulsants, antibiotics, and non-steroidal anti-inflammatory drugs (NSAIDs) being the most common drug classes [3, 46]. The sensitization of the immune system to a causative drug has been described as a T cell-mediated immune response, causing a delayed type VI hypersensitivity reaction. This type of response requires the patient to have a specific HLA allele that codes for a major histocompatibility complex (MHC) that is specific for a drug or drug-metabolite (antigen) [47]. Therefore, certain drug-induced cases of SJS/TEN have been linked to specific HLA haplotypes [47]. Recognition of the MHC-antigen complex by specific T cell receptors results in T cell activation (sensitization). Time from drug exposure to symptom onset is often reported as 3–28 days [39]. All cases in this review reported a minimum of 3 days from drug initiation to rash onset (case specific details of drug duration is reported in Table 2). The exact sequela of immune sensitization in SJS/TEN is still being investigated. Histopathological outcomes from the immune response include subepidermal blisters with widespread necrosis and apoptotic keratinocytes associated with minimal lymphocytic inflammatory infiltrate [1]. It has been observed that there is high expression of granzyme, perforin, and FasL in mononuclear cells in TEN blisters, suggesting CD8 + T-cell activation which is likely responsible for the epidermal keratinocyte necrosis [48, 49].

UVR plays a key role in the pathogenesis of photodistributed SJS/TEN. There are several classifications of UVR based off wavelength: ultraviolet A (UVA) = 320–400 nm, ultraviolet B (UVB) = 290–320 nm, and ultraviolet C (UVC) = 200–290 nm [50, 51]. It is generally accepted that the majority, if not all, of UVC radiation does not make it through the atmosphere to reach the surface of the earth [50]; however, UVB and UVA radiation do make it to the earth surface and, therefore, affect human skin. UVB radiation is readily absorbed by the DNA in the epidermal keratinocytes and as deep as the papillary dermis [52]. UVA radiation is minimally absorbed by the DNA of superficial keratinocytes, but penetrates deep into the reticular dermis where it is absorbed by other molecules and proteins, including drugs that have distributed to the area [52, 53]. More precisely, UVR is electromagnetic radiation that travels from the sun in waves containing photons [52]. These photons are composed of high energy, which are absorbed in the cutaneous layers, that induces electrons to move from their ground state to an unfilled outer electron shell producing an excited state, a high energy state, that is unstable. This is the process that occurs in drug-induced photosensitive reactions [52, 54]. UVA radiation is most commonly responsible for photosensitivity reactions, although UVB may also contribute or be responsible in selected cases [55].

Neither type of photosensitivity reaction (phototoxicity and photoallergy) alone can account for the patient cases in this review. However, all of the causal drugs in this review have been implicated in causing photosensitive reactions [52, 56–80] (Table 5). Thus, when considering the findings of photodistributed rashes that present after UVR exposure in the setting of drug use (drugs known to cause photosensitive reactions), it is feasible that the mechanisms behind drug-induced photosensitive reactions could, at least in part, contribute to photodistributed SJS/TEN.

**Table 5:** Evidence of photosensitive reactions from drugs recognized in causing photodistributed Steven’s–Johnson syndrome and toxic epidermal necrolysis

Drug name	Photosensitivity (non-specific)	Phototoxicity	Photoallergy
Chloroquine ^a^sulfadoxine –pyrimethamine		[56] [52, 57, 58] [60]	[58, 59]
Carbamazepine	[61–63]		
Chlorpromazine	[65]	[57, 66, 67]	[57, 68]
Ciprofloxacin		[52, 57, 58, 65, 69]	
Clobazam		[70]	
Hydroxychloroquine	[65]	[71]	[58, 71]
Ibuprofen	[73, 74]	[52, 57, 58, 65, 72]	
Itraconazole	[75]	[52, 57, 65]	[52, 65]
Lamotrigine	[76]	[77]	
Naproxen sodium		[52, 57, 58, 65, 78]	[58, 59]
Sulfasalazine		[57]	
Tramadol	[80]		

^a^Sulfadoxine is a sulfonamide. Thus, sulfonamide was used to search for photosensitive reactions

A direct drug phototoxicity pathway is one in which UVR induces changes to the drug chemical structure, producing a free radical. The free radicals act on lipids, proteins, and DNA, causing direct damage to cells [81]. An indirect drug phototoxicity pathway is one where the energy from UVR results in reactive oxygen species (ROS). ROS are formed by drug-free radicals reacting with oxygen or excited drug states that allow for the transfer of energy to oxygen via excited triplet cascade [81]. An example of ROS formation in response to UVR was observed when a cancer treatment was evaluated. A combined exposure to cells with UVA irradiation and lomefloxacin caused higher alterations of redox signaling pathways, causing intracellular ROS overproduction and endogenous glutathione depletion in melanoma cells [82]. This had an advantageous effect against melanoma cancer cells. However, excess formation of ROS may occur in healthy tissues, exceeding the body's antioxidant defense mechanisms, allowing for oxidative stress to cause damage to cellular components [83]. The cytotoxic effect of ROS in the skin triggers the immune system to attract T cells to the dermis. In patients with vitiligo, ROS-impaired keratinocytes were shown to mediate CD8 + T cell infiltration [84]. As CD8 + T cells have been shown to be the prominent cells in the pathogenesis of SJS/TEN, it is feasible that the cellular damage caused directly by the photoproduct (a photo-induced drug metabolite), or indirectly through the formation of ROS, triggers immune cells to infiltrate the area. Drug and/or drug photoproducts in the skin recognized as foreign may induce drug sensitization, ensuing the immunological sequelae that results in a clinical presentation of SJS/TEN.

In contrast to phototoxicity, photoallergy is immune mediated: a cell-mediated type IV hypersensitivity reaction. When a drug in the cutaneous tissue absorbs the photons, it is converted into a biologically reactive photoproduct. The photoproduct can then act as a complete antigen or a hapten [57]. The complete antigen model is consistent with the p-I concept, proposing that a drug (or photoproduct) itself is sufficient to bind to MHC and TCR to sensitize the immune system [47, 85]. In the hapten model, the photoproduct binds to protein within the cutaneous tissue (haptenization) to form a complete antigen [57, 86]. Langerhan cells process the antigen and present it to MHC class II molecule and induce the subsequent cell mediated hypersensitivity response, resulting in the homing of activated T-lymphocytes into the skin. As both photoallergy and SJS/TEN reactions are both type IV hypersensitivity reactions that are a result of the immune system responding to a drug, it is reasonable that the immune system could be triggered to go down the SJS/TEN pathway. See 4.4 Differential Diagnoses for more on photosensitive reactions.

An alternate theory is the Koebner phenomenon which describes the appearance of new skin lesions due to trauma, commonly seen in patients with psoriasis and has been postulated as the possible pathophysiology for photodistributed erythema multiforme (PEM) [87]. This theory postulates that UVR causes increased vascular permeability, facilitating the passage of skin antigens into the bloodstream and favoring the formation of circulating antibodies and immune complexes in sun-exposed areas [31, 88]. Thus, UVR precipitated koebnerization leads to a photodistributed pattern [38]. The increased passage of skin antigens may allow for increased susceptibility to the drug/drug metabolites/photoproducts, activating the immune response and causing SJS/TEN.

The next theory is the alterations in intercellular adhesion molecule 1 (ICAM-1) expression. UVR has been shown to induce tumor necrosis factor-alpha (TNF-alpha) secretion by keratinocyte, resulting in increased ICAM-1 expression [22, 89]. Theoretically ICAM-1 may play many roles in the sensitization of the immune system to the drug/drug-photoproduct in photodistributed SJS/TEN. The expression of ICAM-1 by antigen presenting cells (APC), commonly Langerhans cells in the epidermis [90], lowers the concentration of antigen required to activate a naïve T cells into effector and memory T cells [91]. Additionally, ICAM-1 can promote long duration of contact between T cells and APCs [92]. This means that indirectly, via increased ICAM-1 expression, UVR may ultimately play a role in sensitizing the immune system to a low drug dose, precipitating a reaction of SJS/TEN that otherwise may not have happened. This would help explain why three of the reported cases in this review occurred after one dose of a drug [31, 34, 39]. Furthermore, when UVR exposure causes a release of TNF-alpha by keratinocytes, it causes an increased ICAM-I expression by keratinocytes in the epidermis and attracts CD8  + T cells [22, 89, 93]. CD8  + T cells show a clear predominance in epidermis in early stages and through various proposed mechanisms trigger epidermal necrosis [1, 49]. The increased immune response and cell death in UVR exposed skin could explain the photodistributed presentation in these cases.

The last theoretical mechanism is an increase in Fas/FasL expression. Fas ligand (FasL or CD95L) belongs to the TNF family and is responsible for inducing apoptosis in Fas  + cells [94]. Keratinocyte apoptosis is commonly observed in early stages of TEN [49], consistent with large amounts of lytically active FasL that have been shown to be expressed in SJS/TEN [95]. Monoclonal antibodies that interact with Fas/FasL have shown to block the cytological activity, confirming that FasL expression is responsible for the apoptosis that occurs in SJS/TEN [49]. It has been shown that UVR causes FasL upregulation in the epidermis [96]. FasL may be upregulated due to action of TNF-alpha, released by keratinocytes themselves as a result of UVR [89, 97]. Fas receptor expression on keratinocytes has also been shown to increase from both UVB and UVA radiation exposure, with a delay to peak expression at 24 and 12 h, respectively [98]. Upregulation of Fas/FasL in keratinocytes may contribute to the increased involvement of the UVR exposed skin. The activation of the Fas-mediated apoptosis pathway in keratinocytes is presumably activated by toxic drug metabolites [99]. Thus, UVR may increase apoptosis in sun-exposed areas by increasing Fas/FasL expression and creating photoproducts that activate the pathway and further stimulate the immune response in SJS/TEN cases.

### Differential diagnoses

#### Photosensitive reactions

Photosensitivity reactions consist of phototoxic and photoallergic reactions. These reactions are considered the most common type of drug-induced rashes that present in a photodistributed pattern. As the cases in this study appear to present at least partially like drug-induced photosensitive reactions, it is important to distinguish characteristics of phototoxic and photoallergic reactions from photodistributed SJS/TEN.

Phototoxicity is dose dependent and can happen in anyone who ingests (oral) or applies (topical) enough offending agent with concomitant UVR exposure. These responses are unpredictable between people and even repeat exposures [52]. Clinically, they commonly manifest as a severe sunburn with sharp demarcated lines with or without bullous formation. Phototoxic effects generally result in erythema, appearing immediately within minutes to hours, or less commonly a late onset erythema may occur that takes 24–120 h to appear [54]. Generally, these reactions reach a peak of maximal clinical manifestation after 24–48 h of UV exposure [52]. Histopathologically, necrotic keratinocytes are seen along with a predominantly lymphocytic and neutrophilic dermal infiltrate [100].

Photoallergy requires an immunological response that only occurs in sensitized patients. This requires re-exposure of a drug or the continued use of a drug for sufficient time to allow sensitization to occur, reported as three days to years [48]. Most commonly it manifests as an eczematous rash that is less demarcated, often spreading outside of UVR areas. This reaction is not dose dependent; a small amount of drug can trigger the reaction. Symptom onset is generally 24–48 h after UVR exposure [101]. After symptom onset, the peak of maximal clinical manifestation is around 48–72 h [52]. Histopathologic features are identical to those seen in an allergic contact dermatitis, including epidermal spongiosis, vesiculation, exocytosis of lymphocytes, and a perivascular inflammatory infiltrate [100, 102]. Spongiosis represents the histological hallmark of intercellular epidermal edema, corresponding with wide spaces between keratinocytes and elongated intercellular bridges (“spinous processes”), leading to a sponge-like appearance of the epidermis [103]. The most common cutaneous lesion associated with spongiosis is eczematous dermatitis [104], consistent with the eczematous type of rash seen in photoallergic reactions.

Photodistributed SJS/TEN share more commonalities with photoallergic reactions than phototoxic reactions. Photoallergic reactions require sensitization of the immune system, the onset of the rash is generally > 24 h from UVA exposure [22, 29, 30, 34–36, 39], the incidence is rare, they require a low dose of medication [31, 34, 39], and the rash distribution may extend past the sun exposed areas [22, 28, 31–36, 38, 39], although these areas are generally less-affected than sun-exposed areas. No cases in this review report cross-reactivity of related agents. However, cases of drug-induced SJS/TEN due to drug cross-reactivity have been reported in the literature [105–107]. The similarities of photodistributed SJS/TEN, all shown to be photo-drug-induced SJS/TEN, are likely because drug-induced SJS/TEN occurs due to a delayed type IV hypersensitivity reaction, similar to photoallergic reactions [1, 54]. Nevertheless, CD8 + T cells are more prominent in triggering the immune system in SJS/TEN vs. CD4 + T-cells in photoallergy [108]. The pathophysiology of photodistributed SJS/TEN is likely distinct from other drug-induced SJS/TEN cases as UVR plays a role. Nevertheless, it is assumed that a delayed type IV hypersensitivity reaction ultimately takes place as the cases meet diagnostic findings consistent with SJS/TEN.

Findings not observed in a photoallergic or a phototoxic reaction, but that are commonly found in photodistributed SJS/TEN include mucous membrane involvement [22, 28–36, 38, 39], a positive Nikolsky’s sign [22, 30–32, 39], rash involvement of the palmar and plantar surfaces [28, 30, 36, 38, 39]. There is also a unique timeline of maximal clinical manifestation of the rash, reported as 24–48 h in phototoxic reactions, 48–72 h in photoallergic reactions, but commonly progress for days to weeks in photodistributed SJS/TEN [22, 28–33, 36, 38, 39]. The histopathological findings are also distinct. Phototoxic reactions often have necrotic keratinocytes, but less commonly full-thickness epidermal necrosis as in SJS/TEN and the immunological findings are distinct in the dermal/epidermal layers and vesicle fluid (if vesicles are present in the phototoxic reaction) [22, 34, 45, 49]. Although DIF findings are rarely reported in photosensitive reactions, DIF has been reported as positive in photoallergy [109] and negative in phototoxicity [110]. DIF is commonly reported in SJS/TEN and is negative. For additional comparisons see Table 6 [100].

**Table 6:** Differentiating features between drug-induced phototoxic, photoallergic and photodistributed Steven’s–Johnson syndrome and toxic epidermal necrolysis (Adapted from Blakely et al. [100])

UVR reaction type	Phototoxic	Photoallergic	Photodistributed SJS/TEN
Incidence	High	Low	Low
Pathophysiology	Direct tissue injury	Type IV hypersensitive reaction	Type IV hypersensitive reaction^a^
Sensitization Required	No	Yes	Yes
Required dose of medication	High	Low	Low
Cross-reactions to related agents	Low	High	Medium^b^
Agent type	Oral > topical	Oral < topical	Oral > topical
Onset after light exposure	< 24 h (less common > 24 h)	> 24 h	> 24 h
Progression / worsening of rash	24–48 h	48–72 h	> 48 h (up to week(s))
Clinical skin appearance	Exaggerated sunburn	Eczematous / Dermatitis	Photodistributed erythematous macules and flat atypical target lesions with vesicles/bullae and confluence of lesions
Distribution	Only UVR exposed areas	UVR exposed areas; may spread outside UVR areas	UVR exposed areas; may spread outside UVR areas
Palmar and plantar erythema	Uncommon^c^	Uncommon^c^	Common
Mucous membrane involvement	Uncommon^c^	Uncommon^c^	Always
Nikolsky sign	Negative^c^	Negative^c^	Positive
Histology	Necrotic keratinocytes, predominantly lymphocytic and neutrophilic dermal infiltrate	Epidermal spongiosis, exocytosis of lymphocytes and perivascular inflammatory infiltrate	Subepidermal blisters with widespread necrosis (full thickness) and apoptotic keratinocytes associated with minimal lymphocytic inflammatory infiltrate
Direct Immunofluorescence	Negative	May be positive^d^	Negative

*UVR*  Ultraviolet radiation, *SJS*   Steven’s–Johnson syndrome, *TEN*  Toxic epidermal necrolysis

^a^Photo component unknown, but ultimately assumed to result in a drug-induced type IV hypersensitive reaction that is seen in SJS/TEN

^b^No specific study reports the incidence of drug-induced SJS/TEN cross-reactivity, although cases have been reported [105–107]

^c^Not mentioned in the literature as findings of phototoxic/photoallergic reactions

^d^Findings rarely reported [109]

#### Photodistributed erythema multiforme

Photodistributed erythema multiforme (PEM) is rarely reported, but an important differential diagnosis. In one review in 2012, 18 cases of PEM were identified: 10 were drug-induced, four were due to herpes simplex virus, one polymorphous light eruption, and three idiopathic [88]. Erythema multiforme (EM) shares many features with SJS and TEN and all were once considered to be in the same spectrum [111]. Further investigation revealed that EM is considered distinct from SJS/TEN. EM is important to distinguish from SJS/TEN as it is self-limiting with mild or no systemic symptoms and usually resolves without complications, versus a systemic reaction that is potentially life threatening in SJS/TEN [2]. EM is a hypersensitivity reaction that may be caused by drugs but is mostly caused by infections such as herpes simplex virus and mycoplasma [112]. Mucous membrane involvement is only associated with 25–60% of EM cases [113]. EM is characterized by a raised, papular rash with “typical target” lesions with three concentric zones; whereas a flat, macular rash with poorly defined “atypical target” lesions in SJS/TEN generally have two zones and demonstrate confluence of lesions [113, 114]. In SJS/TEN the rashes progress to have more extensive vesicle/bullae formation and are followed by skin sloughing. Palmar and plantar rash can be seen in EM and SJS/TEN. EM is symmetrically distributed on the distal extremities with minimal epidermal detachment, often to 1% or 2% of BSA (< 10% of BSA) [113]. Histologically, the conditions can appear similar in early stages; however, in later stages SJS/TEN can be distinguished from EM: established SJS/TEN shows full-thickness keratinocyte necrosis that develops into subepidermal bullae vs. scattered necrotic keratinocytes that appear in the lower layer of the epidermis (this finding may also be seen in early stages of SJS/TEN) [115]. It has also been noted that the center of a blister in a typical target lesion in EM may also demonstrate full thickness necrosis [116]. Thus, a thorough histological evaluation is warranted. The gravity of the overall clinical presentation, including the involvement of systemic symptoms and the severity of cutaneous and mucosal involvement may suggest SJS/TEN vs. EM early in the patient work-up; however, less severe cases may be less clear and require a detailed work-up to differentiate conditions.

#### Autoimmune bullous diseases, porphyria disorders, and SJS/TEN-Like LE

UVR is known to induce or aggravate autoimmune bullous diseases, including pemphigus foliaceus, pemphigus vulgaris, bullous pemphigoid, and less commonly, linear IgA dermatosis [117]. Bullous diseases have many similarities to drug-induced SJS/TEN. They may present with bullae formation, mucous membrane involvement, a positive Nikolsky’s sign and are widely associated with drug use [13, 118, 119]. Bullous diseases contain autoantibody mediated acantholysis at the basement membrane zone which results in a positive DIF. In contrast to these findings, SJS/TEN is due to necrosis of cells, resulting in the absence of autoantibodies and a negative DIF. Porphyria cutanea tarda and pseudoporphyria are also considered photosensitive bullous diseases that can present in a photodistributed pattern with positive DIF findings that are consistent with the deposition of immunoglobulins and C3 around blood vessels in the dermis and at the dermal epidermal junction [120–122]. Pseudoporphyria is unique in that it is also commonly induced by drugs [122]. Thus, a negative DIF will also help rule out these conditions as potential causes. Furthermore, SJS/TEN-like LE is an important alternate diagnosis that should always be considered, especially in cases that are photodistributed. In addition to a serological autoimmune diagnostic work-up for LE, patients should have a DIF test performed on a biopsy sample of the rash. Patients with SJS/TEN-like LE will have deposition of IgM and IgG in the basement membrane (lupus band) [123]. Additionally, acute cutaneous LE, subacute cutaneous LE, discoid LE, and systemic LE all commonly result in positive DIF of cutaneous samples [124]. In this review, all four cases that reported findings of DIF were negative [22, 32, 36, 38]. DIF should be performed on lesional biopsies in every histopathological workup to help rule out various differential diagnoses that may be photo-drug-induced.

### Distinguishing factors for photodistributed SJS/TEN

The following diagnostic criteria was proposed by the authors to help guide physicians when distinguishing photodistributed SJS/TEN from an alternative diagnosis (Table 7):

**Table 7:** Diagnostic criteria for photodistributed Steven’s–Johnson syndrome and toxic epidermal necrolysis

Must identify = need all 4:
1. A photodistributed rash as a result of UVR exposure after drug initiation (or other known SJS/TEN cause)
2. Rash progression/worsening > 48 h to include vesicles/bullae and/or sloughing of the epidermis
3. Negative direct immunofluorescence
4. More likely SJS/TEN than erythema multiforme
Major criteria (should identify) = need 3/4:
1. A causal drug
2. Mucous membrane involvement
3. Positive Nikolsky sign
4. Histopathology demonstrating full-thickness epidermal necrosis with subepidermal bullae development
Minor criteria (common findings) = need 2/4:
1. Palmar and plantar rash
2. Delayed rash onset that presents no earlier than the day after UVR exposure
3. Satellite lesions/non-discrete borders on UVR protected (clothing covered) skin
4. No flu-like prodrome

*UVR*   Ultraviolet radiation, *SJS*  Steven’s–Johnson syndrome, *TEN*  Toxic epidermal necrolysis

The criteria should continue to be reviewed, and updated when appropriate, with future investigation of photodistributed SJS/TEN. Further elucidation for each criterion is outlined here:

Must identify = need all 4:

1. A photo-distributed rash as a result of UVR exposure after drug initiation (or other known SJS/TEN cause). The timeline of UVR is important as a recall-phenomenon has been reported which likely represents unique pathophysiology [125, 126].
2. Rash progression/worsening that continues for a minimum of 48 h with rash evolution to include vesicles/bullae and/or sloughing of the epidermis. This is a systemic immune disorder that progresses over days to weeks.
3. Negative DIF. If positive, LE, bullous diseases, and porphyria disorders should be further investigated.
4. More likely SJS/TEN than EM. It is important to make this distinction due to mortality risk in SJS/TEN patients. If the disorder is EM, it is self-limited, does not require specialized treatment in a burn center, and the patient is able to perform additional photobiological testing to aid in culprit drug identification: phototesting followed by photopatch testing [88]. Photobiological testing is too risky in SJS/TEN as re-exposure may induce another SJS/TEN case, resulting in a high risk of mortality [1].

Major criteria (should identify) = need 3/4:

1. A causal drug. All cases in this review include a casual drug, while cases without drug involvement have been reported in SJS/TEN-like LE, PEM, bullous diseases, and porphyria disorders.
2. Mucous membrane involvement. Oral, ocular, and/or genital mucositis is reported in 92–100% of SJS patients and nearly all TEN patients, with oral involvement being the most common [113]. It is suspected that the incidence of mucositis would be similar in photodistributed cases as all 12 cases in this review that reported mucous membrane findings reported mucositis.
3. Positive Nikolsky sign. Nearly all should have, but not a “must identify” criteria due to limitations of the test. (See *4.7 case inclusion consideration* for more on Nikolsky sign).
4. Histopathology demonstrating full-thickness epidermal necrosis with subepidermal bullae development. Lesions found in earlier stages of SJS/TEN may not yet have progressed to full-thickness epidermal necrosis, excluding this finding as a “must identify.”

Minor criteria (common findings) = need 2/4:

1. Palmar and plantar rash. No formal report of the incidence could be identified in the literature, although it is commonly reported in SJS/TEN [12] and in the results of this review.
2. Delayed rash onset that presents no earlier than the day after UVR exposure. This was observed in this report and consistent with a delayed hypersensitivity reaction commonly reported in SJS/TEN [1].
3. Satellite lesions/non-discrete borders on UVR protected (clothing covered) skin. These findings were consistent in all cases in this review (those that provided images) to varying degrees.
4. No flu-like prodrome. This finding was consistent with all cases in this review.

Minor criteria 2–4 were important findings in this review that need additional evaluation from further accumulation and investigation of cases to assess their prevalence before they can be considered a “must identify” or “major criteria”. These three findings, in addition to an overall photodistributed rash (number 1 in the “must identify” section), are the findings that are unique from traditional SJS/TEN, summarized as a photodistributed rash with non-discrete borders, occurring at least a day after UVR exposure, and without a flu-like prodrome. The remaining criteria are consistent with traditional SJS/TEN and are important to help distinguish from other photodistributed disorders.

Total % of BSA involvement can help further distinguish between photodistributed SJS, SJS/TEN overlap, and TEN [2]. Although DIF has shown to have significant diagnostic value in LE [123, 124, 127], the negative predictive value of a negative DIF to rule out SJS/TEN-like LE has not been calculated. Thus, physicians should order lupus autoimmune serology to further contribute to LE rule out. Clinical judgment and appropriate diagnostic workup should be included to investigate differential diagnosis previously discussed.

### Treatment

The authors recommend following up to date treatment guidelines for SJS/TEN, emphasizing the importance of a quick diagnosis and transfer to an appropriate care facility, like a burn center [128]. All patients in this study responded well to their treatments and survived, receiving various combinations of systemic steroids, antibiotics, cyclosporine, intravenous immunoglobulin G, and advanced wound care. Patient education should include avoiding causal drugs and mitigating sun exposure with UVR protection when outside.

### Case inclusion consideration

Two cases were less clear about meeting the criteria of disease onset of SJS/TEN after UVR exposure. First, a female with a history of abnormal reactions to ibuprofen chose to take ibuprofen for menstrual cramps which resulted in lip swelling and a minimally reactive rash of small red macules and papules [34]. The rash was stable and non-progressive for two days when the patient decided to self-treat with UVR from a tanning salon that she frequented, triggering the onset of TEN by the following morning [34]. This case was included in the review as the baseline rash was reported to be stable, non-progressive, and radically changed into photodistributed TEN after UVR exposure. The second case was an abstract for a poster. Consequently, it provided fewer details of a female with a history of epilepsy who experienced a case of photodistributed SJS from the ingestion of lamotrigine [37]. This case was also chosen to be included since the photodistribution of the rash implies UVR exposure.

Additionally, cases that reported Nikolsky’s sign findings are worth discussing. A positive Nikolsky’s sign is observed when light pressure is applied to the skin, usually from a clinician’s finger, resulting in the disassociation of the epidermis from the dermis [129]. Five of six cases reported a positive Nikolsky’s sign [22, 30–32, 39], a common reported finding in cases of SJS/TEN [1, 130]. Eloranta et al. [36], presented the only case that reported a negative Nikolsky’s sign. Further evaluation revealed that the histopathologic report of the skin biopsy showed a necrotic epidermis and interface dermatitis with vacuolization and a negative DIF [36], consistent with findings in other cases of SJS/TEN that presented with a positive Nikolsky’s sign [22, 30–32]. The Nikolsky’s sign is a clinical test attempting to evaluate if the epidermal cells have detached from the dermis which was suggested in the biopsy findings in this case. Obtaining a positive Nikolsky’s sign depends on correctly performing and interpreting the findings. One case showed that although 75% of a patient’s BSA presented with a rash consistent with TEN, the Nikolsky’s sign was only positive in 9% of BSA upon presentation, which progressed to 20% when re-evaluated 24 h later [107]. Thus, if a Nikolsky’s sign is only performed on part of the rash, a false negative may result. Additionally, the timing of the test matters. Tests performed later in the disease process are more likely to be positive. This correlated with histological findings that show progression from individual keratinocyte apoptosis to full thickness necrosis in later stages. Due to variability in Nikolsky’s sign findings, along with the histopathologic report and other clinical findings that are consistent with SJS, the case published by Eloranta et al. [36] was still included in the analysis.

### Additional radiation-related considerations in SJS/TEN Cases

Several studies fell short of meeting inclusion criteria but are worth discussing. A case of a 67-year-old female with SJS/TEN overlap and a positive Nikolsky’s sign reported that sun exposure was a potentiating factor, providing images of the patient’s rash with photo-demarcated borders [10]. This case was excluded due to limited case information (only description was in a figure legend), and no causal drug or timeline of sun exposure was provided. Two studies reported patients with past histories of psoriasis who regularly received treatments of ultraviolet-B light and experienced fatal cases of TEN, one after seven days on cefozopran and the other 16 days after starting etretinate [131, 132]. These cases did not meet inclusion criteria because the studies did not report whether the patient received ultraviolet-B light therapy after starting the causal drug. Three additional cases of SJS/TEN, which reported patients who received methoxsalen with concomitant UVR for the treatment of vitiligo or psoriasis [133], were excluded for similar reasons.

There are also reports of UVR exposure preceding introduction of the causal drug that resulted in SJS/TEN presentations. A patient who experienced a bullous sunburn on his back and thighs presented seven months later with a case of TEN after taking trimethoprim-sulfamethoxazole; his rash replicated the distribution of the previous sunburn [125]. Another study reported a man with a history of a left forearm sarcoma resection and treated with a split-thickness skin graft and radiotherapy who presented with SJS several months later [126]. His rash was localized primarily to the radiation exposure site following a 10-day regimen of trimethoprim-sulfamethoxazole for an infection. These were considered recall-like reactions as the areas of radiation exposure expressed significantly more vesiculobullous involvement than other areas of the body [126]. A recall reaction occurs after ionizing or ultraviolet radiation damages the skin, permanently destabilizing the skin’s immune behavior, leaving the affected skin compromised and vulnerable to subsequent immune-related disorders [134]. Recall reactions likely have a different pathogenesis and thus are considered a distinct diagnosis from photodistributed SJS/TEN cases that are described in this review.

Radiotherapy has also been reported to cause drug-induced SJS/TEN presentations after the initiation of a new drug regimen, similar to the UVR cases described in this review. One such case of SJS was reported after a patient received concomitant phenobarbital and radiation therapy, which resulted in rash distribution limited to the radiation exposure areas [135]. The same study recognizes an additional 21 cases of atypical erythema multiforme, TEN, and SJS in patients receiving radiation therapy while on concomitant phenytoin, phenobarbital, or carbamazepine. Additional studies described SJS/TEN overlap from concurrent gemcitabine or temozolomide and radiotherapy [136, 137]. These cases were not included as all cases did not adequately describe rash onset, distribution, and type of radiation exposure. Future cases of SJS/TEN that are precipitated by radiotherapy should be evaluated by the proposed diagnostic criteria to determine if they are photodistributed SJS/TEN.

### Limitations and future directions

There were many studies that met inclusion criteria. However, one limitation to this review is the level of detail provided by the individual studies, thus limiting the analyses and comparisons that could be performed. Furthermore, it is difficult to ascertain the prevalence of photodistributed SJS/TEN. In the literature, UVR’s role in SJS/TEN cases is not widely documented. Due to the minimal coverage, it is plausible that many UVR-linked cases have gone unrecognized or unreported.

Future directions should focus on further investigation of the pathophysiology that is responsible for photodistributed SJS/TEN, as the current understanding is theoretical.

## Conclusion

Physicians should be aware that UVR may precipitate SJS/TEN in patients taking susceptible drugs. Rash onset occurs after UVR exposure in a photodistributed pattern, with a delay of at least 24 h. As the rash progresses for at least 48 h, vesiculobullous lesions with skin sloughing form. The photodistributed pattern remains with non-discrete borders and satellite lesions on UVR protected (clothing covered) skin. A lack of a flu-like prodrome is also unique from traditional cases of SJS/TEN. Additionally, findings of mucositis, palmar and plantar rash, a positive Nikolsky sign, and a prolonged disease course can help distinguish photodistributed SJS/TEN from photosensitive reactions. Similarly, a negative DIF can help rule out other photo-induced autoimmune bullous diseases, porphyria disorders, and SJS/TEN-like LE. Photodistributed SJS/TEN appears to be photo-drug-induced with a unique onset and rash presentation that should be recognized as a distinct diagnosis.

## Supplementary Information

**Additional file1:** A log of individual searches with their corresponding results to identify photodistributed cases of SJS/TEN.

## Data Availability

Not applicable.

## Abbreviations

ALDEN: Algorithm of drug causality for epidermal necrolysis
BASE: Bielefeld academic search engine
CAT: Causality assessment tools
EM: Erythema multiforme
ICAM-1: Intercellular adhesion molecule 1
LE: Lupus erythematosus
MHC: Major histocompatibility complex
NTIS: National technical information service
PEM: Photodistributed erythema multiforme
ROS: Reactive oxygen species
SSRN: Social science research network
SJS: Stevens-Johnson syndrome
TEN: Toxic epidermal necrolysis
TNF-alpha: Tumor necrosis factor-alpha
UVA: Ultraviolet A
UVB: Ultraviolet B
UVC: Ultraviolet C
UVR: Ultraviolet radiation
