# Supplementary material for: Photodistributed Stevens–Johnson syndrome and toxic epidermal necrolysis: a systematic review and proposal for a new diagnostic classification
Source: Eur J Med Res. 2023 Jun 12;28:188. doi: 10.1186/s40001-023-01142-2 (PMC10259004; doi:10.1186/s40001-023-01142-2)
Supplement: Supplementary file 1 — Additional file1: A log of individual searches with their corresponding results to identify photodistributed cases of SJS/TEN. [file 40001_2023_1142_MOESM1_ESM.docx]

| PubMed (searched 09/09/2021) |  |  |  |  |
| --- | --- | --- | --- | --- |
| Searched Criteria | Total results | Full articles screened | Articles included in study | Reference #: |
| “stevens-johnson syndrome” AND ultraviolet | 11 | 5 | 2 | 34,38 |
| “stevens-johnson syndrome” AND “photo-distributed” | 1 | 1 | 1 | 36 |
| “stevens-johnson syndrome” AND “photo-induced" | 6 | 6 | 5 | 22,30,31,32,35 |
| “stevens-johnson syndrome” AND photosensitivity | 84 | 14 | 6 | 22,28^c^,29^c^,35,36,38 |
| “stevens-johnson syndrome” AND photo | 15 | 9 | 7 | 22,30,31,32,33,35,36 |
| “toxic epidermal necrolysis” AND ultraviolet | 7 | 3 | 2 | 34,38 |
| “toxic epidermal necrolysis” AND “photo-distributed" | 0 | 0 | 0 |  |
| “toxic epidermal necrolysis” AND “photo-induced” | 4 | 4 | 3 | 22,31,32 |
| “toxic epidermal necrolysis” AND photosensitivity | 39 | 10 | 2 | 22,38 |
| “toxic epidermal necrolysis” AND photo | 10 | 6 | 4 | 22,31,32,33 |
| Google Scholar (https://scholar.google.com/ searched 09/10/2021) |  |  |  |  |
| Search Criteria (screened up to 150 results for each search) | Total results | Full articles screened | Articles included in study | Reference #: |
| “stevens-johnson syndrome” AND ultraviolet | 5190 (150 screened) | 30 | 2 | 30,31 |
| “stevens-johnson syndrome” AND “photo-distributed” | 95 | 21 | 3 | 30,31,38 |
| “stevens-johnson syndrome” AND “photo-induced" | 186 | 18 | 5 | 30,31,32,33,35 |
| “stevens-johnson syndrome” AND photosensitivity | 4920  (150 screened) | 24 | 4 | 22,32,33,34 |
| “stevens-johnson syndrome” AND photo | 3970  (150 screened) | 28 | 6 | 22,30,31,32,35,36 |
| “toxic epidermal necrolysis” AND ultraviolet | 5090  (150 screened) | 26 | 4 | 22,31,32,38 |
| “toxic epidermal necrolysis” AND “photo-distributed" | 88 | 6 | 3 | 30,31,38 |
| “toxic epidermal necrolysis” AND “photo-induced” | 195  (150 screened) | 17 | 7 | 22,30,31,32,33,35,38 |
| “toxic epidermal necrolysis” AND photosensitivity | 4470  (150 screened) | 6 | 2 | 33,38 |
| “toxic epidermal necrolysis” AND photo | 3220  (150 screened) | 7 | 3 | 22,32,34 |
| TRIP medical database (searched 09/10/2021) |  |  |  |  |
| Searched Criteria | Total results | Full articles screened | Articles included in study | Reference #: |
| “stevens-johnson syndrome” AND ultraviolet | 65 | 10 | 0 |  |
| “stevens-johnson syndrome” AND “photo-distributed” | 0 | 0 | 0 |  |
| “stevens-johnson syndrome” AND “photo-induced” | 18 | 3 | 0 |  |
| “stevens-johnson syndrome” AND photosensitivity | 85 | 6 | 0 |  |
| “stevens-johnson syndrome” AND photo | 53 | 3 | 0 |  |
| “toxic epidermal necrolysis” AND ultraviolet | 67 | 7 | 0 |  |
| “toxic epidermal necrolysis” AND “photo-distributed" | 1 | 0 | 0 |  |
| “toxic epidermal necrolysis” AND “photo-induced” | 20 | 2 | 1 | 32 |
| “toxic epidermal necrolysis” AND photosensitivity | 86 | 4 |  |  |
| “toxic epidermal necrolysis” AND photo | 37 | 2 | 1 | 32 |
| USA.gov (https://www.usa.gov/ searched 09/15/2021) |  |  |  |  |
| Searched Criteria  (Database did not report total results for each search, first 150 results were screened) | Total results | Full articles screened | Articles included in study | Reference #: |
| “stevens-johnson syndrome” AND ultraviolet | 150+  (150 screened) | 4 | 1 | 38 |
| “stevens-johnson syndrome” AND “photo-distributed” | 4 | 5 | 2 | 36,38 |
| “stevens-johnson syndrome” AND “photo-induced" | 18 |  | 6 | 30,31,32,35,34,38 |
| “stevens-johnson syndrome” AND photosensitivity | 150+  (150 screened) | 3 | 1 | 22 |
| “stevens-johnson syndrome” AND photo | 150+  (150 screened) | 4 | 2 | 30,36 |
| “toxic epidermal necrolysis” AND ultraviolet | 150+  (150 screened) | 4 | 1 | 38 |
| “toxic epidermal necrolysis” AND “photo-distributed" | 4 | 3 | 1 | 38 |
| “toxic epidermal necrolysis” AND “photo-induced” | 147 | 8 | 4 | 31,32,33,38 |
| “toxic epidermal necrolysis” AND photosensitivity | 150+  (150 screened) | 4 | 2 | 33,38 |
| “toxic epidermal necrolysis” AND photo | 150+  (150 screened) | 6 | 3 | 31,32,33 |
| Bielefeld Academic Search Engine (BASE) (https://www.base-search.net/ searched 09/15/2021) |  |  |  |  |
| Searched Criteria | Total results | Full articles screened | Articles included in study | Reference #: |
| “stevens-johnson syndrome” AND ultraviolet | 5 | 1 | 0 |  |
| “stevens-johnson syndrome” AND “photo-distributed” | 3 | 3 | 2 | 36,37^d^ |
| “stevens-johnson syndrome” AND “photo-induced" | 6 | 6 | 3 | 30,35,36 |
| “stevens-johnson syndrome” AND photosensitivity | 36 | 7 | 1 | 38 |
| “stevens-johnson syndrome” AND photo | 12 | 8 | 4 | 30,35,36,37^d^ |
| “toxic epidermal necrolysis” AND ultraviolet | 3 | 1 | 0 |  |
| “toxic epidermal necrolysis” AND “photo-distributed" | 0 | 0 | 0 |  |
| “toxic epidermal necrolysis” AND “photo-induced” | 6 | 2 | 2 | 22,32 |
| “toxic epidermal necrolysis” AND photosensitivity | 37 | 4 | 2 | 38,35 |
| “toxic epidermal necrolysis” AND photo | 12 | 4 | 2 | 22,32 |
| Qinsight (searched 09/15/2021) |  |  |  |  |
| Searched Criteria^a^  (Screened up to 150 results for each search) | Total results | Full articles screened | Articles included in study | Reference #: |
| “stevens-johnson syndrome” AND ultraviolet | 1169  (150 screened) | 3 | 1 | 38 |
| “stevens-johnson syndrome” AND “photo-distributed” | 206  (150 screened) | 4 | 1 | 36 |
| “stevens-johnson syndrome” AND “photo-induced" | 302  (150 screened) | 4 | 3 | 22,30,38 |
| “stevens-johnson syndrome” AND photosensitivity | 5633  (150 screened) | 0 | 0 |  |
| “stevens-johnson syndrome” AND photo | 312  (150 screened) | 0 | 6 | 22,30,31,32,35,36 |
| “toxic epidermal necrolysis” AND ultraviolet | 2277  (150 screened) | 4 | 2 | 34,38 |
| “toxic epidermal necrolysis” AND “photo-distributed" | 302  (150 screened) | 1 | 0 |  |
| “toxic epidermal necrolysis” AND “photo-induced” | 417  (150 screened) | 8 | 5 | 22,30,31,32,35 |
| “toxic epidermal necrolysis” AND photosensitivity | 6184  (150 screened) | 4 | 2 | 30,33 |
| “toxic epidermal necrolysis” AND photo | 430  (150 screened) | 9 | 6 | 22,30,31,32,36,38 |
| Medicine Case Reports and Study Protocols (Searched 12/29/2021) |  |  |  |  |
| Searched Criteria | Total results | Full articles screened | Articles included in study | Reference #: |
| All search combinations | 1 | 1 | 1 | 39 |
| Medscape (https://www.medscape.com/ searched 09/16/2021) |  |  |  |  |
| Searched Criteria | Total results | Full articles screened | Articles included in study | Reference #: |
| “stevens-johnson syndrome” AND ultraviolet | 24 | 2 | 0 |  |
| “stevens-johnson syndrome” AND “photo-distributed” | 3 | 1 | 0 |  |
| “stevens-johnson syndrome” AND “photo-induced" | 2 | 1 | 0 |  |
| “stevens-johnson syndrome” AND photosensitivity | 127 | 1 | 0 |  |
| “stevens-johnson syndrome” AND photo | 22 | 2 | 0 |  |
| “toxic epidermal necrolysis” AND ultraviolet | 30 | 2 | 0 |  |
| “toxic epidermal necrolysis” AND “photo-distributed" | 6 | 0 | 0 |  |
| “toxic epidermal necrolysis” AND “photo-induced” | 12 | 0 | 0 |  |
| “toxic epidermal necrolysis” AND photosensitivity | 119 | 2 | 0 |  |
| “toxic epidermal necrolysis” AND photo | 15 | 0 | 0 |  |
| Searched Criteria (only searches that produced results were reported) | Total results | Full articles screened | Articles included in study | Reference #: |
| OECD (searched 09/13/2021)^b^ |  |  |  |  |
| “toxic epidermal necrolysis” AND ultraviolet | 1 | 0 | 0 |  |
| ClinicalTrials.org (searched 09/13/2021)^b^ |  |  |  |  |
| “stevens-johnson syndrome” AND ultraviolet | 1 | 0 | 0 |  |
| Sigma Repository (searched 09/13/2021)^b^ |  |  |  |  |
| “stevens-johnson syndrome” AND ultraviolet | 5 | 2 | 0 |  |
| “toxic epidermal necrolysis” AND “photo-induced” | 3 | 3 | 0 |  |
| “toxic epidermal necrolysis” AND photo | 1 | 1 | 0 |  |
| OAIster: Find the Pearls (searched 09/13/2021)^b^ |  |  |  |  |
| “stevens-johnson syndrome” AND photosensitivity | 7 | 2 | 0 |  |
| “toxic epidermal necrolysis” AND “photo-induced” | 1 | 1 | 0 |  |
| “toxic epidermal necrolysis” AND photosensitivity | 7 | 1 | 0 |  |
| "toxic epidermal necrolysis" AND photo | 4 | 0 | 0 |  |
| Cochrane Library (searched 09/14/2021)^b^ |  |  |  |  |
| “stevens-johnson syndrome” AND photosensitivity | 3 | 0 | 0 |  |
| “stevens-johnson syndrome” AND photo | 3 | 0 | 0 |  |
| “toxic epidermal necrolysis” AND photosensitivity | 7 | 0 | 0 |  |
| New York Academy of Medicine Grey Literature Report (searched 09/13/2021)^b^ |  |  |  |  |
| Social Science Research Network (SSRN) (searched 09/13/2021)^b^ |  |  |  |  |
| Grey Matters: a practical search tool for health-related grey literature (https://www.cadth.ca/grey-matters-practical-tool-searching-health-related-grey-literature-0/ searched 09/13/2021)^b^ |  |  |  |  |
| National Technical Information Service (NTIS) (searched 09/13/2021)^b^ |  |  |  |  |
| UpToDate (https://www.uptodate.com/  searched 9/16/2021) |  |  |  |  |
| Searched: https://www.uptodate.com🡪Stevens-Johnson syndrome and toxic epidermal necrolysis: Pathogenesis, clinical manifestations, and diagnosis🡪RISK FACTORS🡪Other factors | 3 | 3 | 0 |  |
| Total results (including repeats): 46864  Total articles screened (including repeats): 5147  Total full-text articles screened (including repeats): 416  Total articles included (excluding repeats): 13 | | | | |

Additional File 1 A Log of Individual Searches with Their Corresponding Results to Identify Photodistributed Cases of SJS/TEN

^a^Focused searches were first reviewed before broader results. As focused results were fewer and none identified unique articles to be included in the study, only broader results were computed in the analysis.

^b^All ten searches were run for all databases, registries/repositories, websites/web search engines. Only searches that produced results were reported in the table. (Ten searches: “stevens-johnson syndrome” AND ultraviolet, “stevens-johnson syndrome” AND “photo-distributed,” “stevens-johnson syndrome” AND “photo-induced,” “stevens-johnson syndrome” AND photosensitivity, “stevens-johnson syndrome” AND photo, “toxic epidermal necrolysis” AND ultraviolet, “toxic epidermal necrolysis” AND “photo-distributed,” “toxic epidermal necrolysis” AND “photo-induced,” “toxic epidermal necrolysis” AND photosensitivity, “toxic epidermal necrolysis” AND photo)

^c^Articles found *only in* PubMed

^d^Articles found *only outside* of PubMed
